# Does opening a milk bank in a neonatal unit change infant feeding practices? A before and after study

**DOI:** 10.1186/1746-4358-5-4

**Published:** 2010-03-08

**Authors:** María Isabel Utrera Torres, Carmen Medina López, Sara Vázquez Román, Clara Alonso Díaz, Jaime Cruz-Rojo, Elisa Fernández Cooke, Carmen R Pallás Alonso

**Affiliations:** 1 Service of Neonatology, Hospital Universitario 12 de Octubre, Avenida de Córdoba s/n, 28041, Madrid, Spain; 2 Department of Pediatrics, Hospital Universitario 12 de Octubre, Avenida de Córdoba s/n, 28041, Madrid, Spain

## Abstract

**Background:**

Donor human milk banks are much more than simple centers for collection, storage, processing, and distribution of donor human milk, as they cover other aspects and represent a real opportunity to promote and support breastfeeding. The aim of our study is to assess the impact that opening a human milk bank has had on the proportion of infants receiving exclusive breast milk at discharge and other aspects related to feeding children with birth weight < or = 1500 g or < 32 weeks gestation admitted to the neonatal unit.

**Methods:**

The study included babies of < or = 1500 g or < 32 weeks gestation. Fifty infants born from February to July in 2006, before the opening of the human milk bank, and 54 born from February to July in 2008, after its opening, met inclusive criteria. We collected data about days of hospital stay, hours of life when feeding was started, hours of life when full enteral feeding was attained, the type of milk received during admission, and the type of feeding on discharge.

**Results:**

Children born in 2008 commenced feeding 16 hours earlier than those born in 2006 (p = 0.00). The proportion of infants receiving exclusive breast milk at discharge was 54% in 2006 and 56% in 2008 (p = 0.87). The number of days they received their mother's own milk during the first 28 days of life was 24.2 days in 2006, compared to 23.7 days in 2008 (p = 0.70). In 2006, 60% of infants received infant formula at least once in the first 28 days of life, compared to 37% in 2008 (p = 0.01).

**Conclusions:**

The opening of a donor human milk bank in a neonatal unit did not reduce the proportion of infants exclusively fed with breast milk at discharge, but did reduce the proportion of infants that received infant formula during the first four weeks of life. Also, having donor human milk available enables commencement of enteral feeding earlier.

## Background

Breastfeeding is the normal way to feed infants by providing them the nutrients they need for healthy growth and development [1-6]. Breastfeeding also facilitates the attachment between mother and child, as it requires physical contact and interaction [7]. Artificial feeding is an important risk factor for infant morbidity and mortality, especially for premature children [8,9]. However, mothers do not always have enough breast milk available to feed premature children. In these cases, donor human milk is the best alternative [1]. The benefits of using donor human milk for premature and sick infants in the neonatal intensive care unit are well known. Evidence has demonstrated that donor human milk protects against necrotizing enterocolitis [2-4,10-12] and infections [13] during the neonatal period. Long-term benefits have also been shown, such as enhanced psychomotor developmental indices [14] or reduced cardiovascular risk factors [15].

The donor human milk bank of the Neonatology Department of Hospital 12 de Octubre in Madrid was inaugurated on 17^th ^December 2007. It is the second milk bank operating in Spain, after the milk bank in the Balearic Islands, and the first one to be opened in a neonatal unit. The usual recipients of donor milk are premature children < 32 weeks gestation or those weighing < 1500 grams, when their mother's milk supply is inadequate to meet the baby's needs. Other potential recipients are infants with feeding intolerance or surgical patients with short gut syndrome.

Before the opening of the milk bank, one of the possible problems to be considered was if the neonatologists would be as supportive as they were before encouraging mothers to breastfeed, once they knew they had human milk at their disposal. On the other hand it could be the mothers who, knowing about the bank, found themselves less motivated to do frequent extractions and all the effort required to breastfeed their children. Therefore, the aim of this study is to assess the impact that opening a milk bank has had on the proportion of infants receiving exclusive breast milk at discharge and other aspects related to feeding children with birth weight < or = 1500 g or < 32 weeks gestation admitted to the neonatal unit. And also whether enteral feeding was started earlier and was fully attained in less time with donor human milk rather or with infant formula.

## Methods

**Figure 1:**
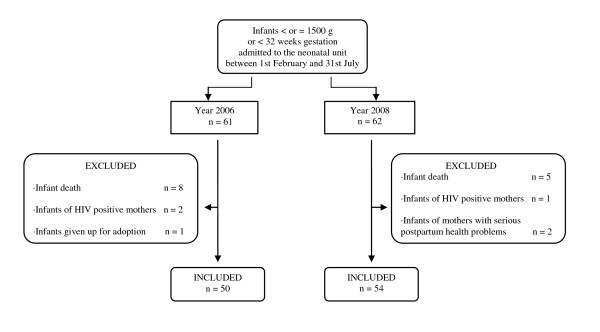
**Participant flow in the study**.

We included newborns born in our hospital, with birth weight < or = 1500 g or < 32 weeks gestational age, between 1st February and 31st July of 2006 and in the same period in 2008, when the milk bank was already working. In 2006 and 2008, 61 and 62 infants with these characteristics were respectively admitted. Children whose mothers had no chance to breastfeed due to serious postpartum health problems, HIV infection or because the child was given in adoption, were excluded (3 cases in each period). In 2006, 8 infants (13%) died before discharge and 5 (8%) in 2008. Finally, 50 infants from 2006 and 54 from 2008 were included in the study (Figure 1). The aim of the study was to evaluate the foundation of a human milk bank in a neonatal unit by a before and after study design. In our unit we have concurrent data about the evolution of the infants with birth weight < or = 1500 g or < 32 weeks gestation age, in this study we used the already collected information. The 1st February was selected as the starting date because it is when the bank was fully operating.

For children born in 2006, the enteral feeding administered was their mother's own milk, infant formula or both. In 2008, in addition to the mother's own milk and/or infant formula, donor human milk was also used. When the milk bank was starting up there were few milk reserves, nevertheless, all the children with weight < or = 1500 g or < 32 weeks gestation age were fed with donor milk, at least during the first four weeks of life, if they did not have their own mother's milk or if this was insufficient. Infants received parenteral feeding until full enteral feeding was attained. The criterion for discharge from the unit has not been modified over these years.

We collected data about the following variables: maternal age, previous pregnancies, type of birth, gender, age at gestation, and birth weight. Days of hospital stay, hours of life when enteral feeding was started, hours of life when full enteral feeding was attained, the type of milk received during admission, and the type of feeding on discharge (referring to the type of feeding that they received in the last 48 hours before discharge) were considered as result variables.

We define "exclusive breastfeeding" and "exclusive breast milk" as feeding babies with no other food or liquid, not even water, with the exception of drops or syrups consisting of vitamins, mineral supplements or medicine, apart from breast milk. Besides, we define "partial breastfeeding" and "any breast milk" as feeding infants with breast milk and other sources of energy and nutrients, like an addition of nutritional supplements for human milk. When we talk about "breastfeeding" and "breast milk" the term covers any kind of breastfeeding/breast milk feeding with or without infant formula or other infant food. During their stay in the neonatal unit, the babies were fed directly from the breast, received their own mother's milk or donor milk by tube or by syringe according to their abilities to feed.

The mothers provided written consent so data for research could be used. The Institutional Review Board of the study centre approved the recruitment methods, the consent process and the study protocol.

A descriptive statistical analysis was performed. Central tendency and dispersion measurements were calculated. For the analytical study, associations were estimated between the different variables collated for the two groups (2006, 2008). A statistically significant association was considered when p < 0.05. The statistical test used was the *t *test and Wilcoxon test for comparison of means and chi-square test and exact Fisher test for categorical variables. The statistical program SAS software (SAS Institute Inc, Cary NC) was used to perform the analysis. Quantitative variables were presented as mean and standard deviation. The relative frequency of the categorical variables were calculated and represented as percentages. The main purpose of the present study was to compare the proportion of infants receiving exclusive breast milk at hospital discharge before and after the opening of the milk bank.

## Results

The characteristics of mothers and infants from 2006 and 2008 are compared in Table 1. Regarding the type of birth, 66% of the children included in the study from 2006 and 70% from 2008 (p = 0.63) were born by caesarean section and 50% and 59% in 2006 and 2008 respectively, were males. Table 2 shows the proportion of children who received different types of feeding during the first 28 days of life in 2006 and 2008. No child received exclusive infant formula in either 2006 or 2008. Thirty infants (60%) in 2006 and 20 (37%) in 2008 received formula at some time during the first 28 days of life (p = 0.01). Table 3 shows the results referring to hours of life until commencing feeding, hours of life when full enteral feeding was attained, and the days fed with the mother's own milk during the first 28 days of life. Twenty-seven (54%) and 30 (56%) infants, received exclusive breast milk 48 hours before discharge (p = 0.87) while 43 (86%) and 42 (78%) received "any breast milk" upon discharge (p = 0.27), in 2006 and 2008 respectively.

**Table 1:** Characteristics of mothers and neonates

	2006 (n = 50)		2008 (n = 54)		
	Mean ± standard deviation	Range	Mean ± standard deviation	Range	p-value
Maternal age (y)	28.4 ± 6.4	15 - 41	31.6 ± 5.9	16 - 44	0.01
Previous pregnancy	0.8 ± 1.2	0 - 5	0.8 ± 1.0	0 - 4	1.00
Gestational age (w)	30.9 ± 2.7	25 - 36	30.2 ± 2.2	25 - 35	0.16
Weight of neonate (g)	1285 ± 297	675 - 2120	1291 ± 351	670 - 2210	0.92
Days admission	48.1 ± 22.2	18 - 112	50.6 ± 24.0	17 - 108	0.58
Corrected age at time of discharge (w)	37.7 ± 2.5	33.3-46.9	37.4 ± 2.2	33.7-43.9	0.50

Values are expressed as mean, standard deviation and range.

**Table 2:** Type of milk received during first 28 days of life

	2006		2008	
	**Number of children** **(n = 50)**	**%**	**Number of children** **(n = 54)**	**%**

^1 ^Exclusively mother's own milk	20	40	7	13
^2 ^Mother's own milk + donor milk	0	0	27	50
^3 ^Mother's own milk + infant formula	30	60	2	4
^4 ^Mother's own milk + donor milk + infant formula	0	0	18	33
^5 ^Exclusively infant formula	0	0	0	0
^6 ^Fortified	15	30	7	13

^1 ^Infants fed only with mother's own milk directly from the breast or by tube or syringe.

^2 ^Infants who received donor milk besides mother's own milk.

^3 ^Infants who received infant formula besides mother's own milk.

^4 ^Infants who received donor human milk and infant formula besides mother's own milk.

^5 ^Infants fed only with infant formula.

^6 ^Addition of a nutritional supplement for human milk.

**Table 3:** Commencement of enteral feeding, full enteral feeding, and days with mother's own milk

	2006 (n = 50)		2008 (n = 54)		
	**Mean ± standard deviation**	**Range**	**Mean ± standard deviation**	**Range**	**p-value**

Hours of life at commencement of EF	42.9 ± 26.4	9 - 137	26.6 ± 22.4	3 - 148	0.00
Hours of life at full EF	229.3 ± 190.2	58 - 1235	185.1 ± 117.8	61 - 713	0.16
^1 ^Days with mother's own milk during 28 days of life	24.2 ± 5.5	7 - 29	23.7 ± 7.3	2 - 29	0.70

Values are expressed as mean, standard deviation and range.

Abbreviation: EF: enteral feeding.

^1 ^Days that infants received mother's own milk, either exclusively or partially, directly from the breast or by tube or syringe, during the first 28 days of life.

## Discussion

In premature children < or = 1500 g or < 32 weeks gestation, feeding with donor human milk, used as a milk substitute for mother's own milk when it is not available or as an additional supply when mother's own milk is not enough, has not led to a decrease in the proportion of children exclusively fed with breast milk upon discharge from hospital. The total number of days that premature children received their mother's own milk during the first four weeks of life is similar in both time periods. The availability of donor milk has reduced by 23% the proportion of children who receive infant formula at some time during their admission in our neonatal unit.

The percentage of infants receiving exclusive breast milk was reduced from 40% to 13%, because in 2008 donor milk was used when there was not enough milk from their own mothers, whereas in 2006, the infants were fed by parenteral nutrition the first days of their lives to avoid infant formula.

The use of donor milk has dropped the age of commencing enteral feeding by 16 hours. Neonatologists at Hospital 12 de Octubre have always tried to introduce enteral feeding as early as possible, but in very preterm infants whose mothers were willing to breastfeed, they waited for the mother to have enough breast milk to start feeding in order to avoid exposure to infant formula during the first days of life. Now donor human milk is available, this delay is avoided and enteral feeding is started earlier.

This study has had an early onset in order to determine whether the opening of a donor human milk bank is having a negative effect on the proportion of infants receiving exclusive breast milk upon discharge. The objective of our study was to make a self-evaluation of the department and to assess how the professionals work in the promotion and support of breastfeeding in mothers of very premature children. The fact that enteral feeding can be started earlier probably leads to a reduction in the number of hours with a central venous catheter and therefore to a possible reduction in the risk of infection [16,17]

The rates of breastfed children vary according to the different countries regarded. World Health Organization estimates that only 35% of the infants from all over the world are exclusively breastfed during the first four months of their lives [1]. These low rates are really worrying so different countries are adopting measures, such as The United States in the program "Healthy People 2010" [18], which sets the objective to increase the proportion of mothers who breastfeed their infants to 75% in the early postpartum period and to 50% during at least six months postpartum. There are studies that show that there are higher rates of breastfed children among those who have to be interned in a neonatal unit [19,20]. However, breastfeeding in preterm babies is more difficult because of their impossibility to feed directly from the breast and therefore they are less likely to receive breast milk than term infants [21].

The proportion of infants exclusively fed with breast milk upon hospital discharge in very premature children in the Hospital 12 de Octubre neonatology department is approximately 55% for exclusive breast milk and 80% if we include any breast milk. Both remained constant throughout the study period. These results are satisfactory; however, we have to offer the same support to mothers who decide to breastfeed their premature children with their own milk, despite having donor milk available [20].

Donor human milk banks are much more than simple centers for the collection, storage, processing, and distribution of donor human milk, as they cover other aspects and represent a real opportunity to promote and support breastfeeding. The donor human milk bank of Hospital 12 de Octubre works to promote and support breastfeeding and it also encourages the families to follow the kangaroo method, a mother care method that promotes skin to skin on the mother's chest and therefore facilitates breastfeeding [22]. The revision of the number of babies exclusively fed with breast milk, as we have taken into account in this study, helps to attain the objective of implementing the baby-friendly hospital initiative proposed by UNICEF in our maternal-children's hospitals [23].

## Conclusions

There is very little information on the impact of the opening of a human milk bank in a neonatal unit and how this affects the proportion of children receiving exclusive breast milk at discharge. We believe that this study--in spite of its limitations--is of interest to other units which plan to open a donor human milk bank. We demonstrate how milk banks do not cause reduction in the rates of breast milk feeding. Besides, they help to reduce the proportion of infants that receive infant formula in the first weeks of life and allow them to attain full enteral feeding as soon as possible.

## Competing interests

The authors declare that they have no competing interests.

## Authors' contributions

MIUT had primary responsibility for developing the study and writing the manuscript. CML participated in the interpretation of data and writing the manuscript. SVR and CAD helped to develop the study. JC-R performed statistical analysis. EFC participated in the revision and language correction of the manuscript. CRP participated in the design of the study and wrote and revised the manuscript. All authors approved the final manuscript.

## Authors' Information

MIUT, CML, SVR, CAD and CRP are all affiliated with Red SAMID (ref RD008/0072).
